# A Novel Sustainable Process for Multilayer Graphene Synthesis Using CO_2_ from Ambient Air

**DOI:** 10.3390/ma15175894

**Published:** 2022-08-26

**Authors:** Matthew Colson, Leandro Alvarez, Stephanie Michelle Soto, Sung Hee Joo, Kai Li, Andrew Lupini, Kashif Nawaz, Ignatius Fomunung, Mbakisya A. Onyango, Michael K. Danquah, Joseph Owino, Sungwoo Yang

**Affiliations:** 1 Department of Civil and Chemical Engineering, University of Tennessee at Chattanooga, Chattanooga, TN 47431, USA; 2 Ecophene Inc., Chattanooga, TN 47405, USA; 3 Energy Science and Technology Directorate, Oak Ridge National Laboratory, Oak Ridge, TN 37831, USA; 4 Center for Nanophase Materials Sciences, Oak Ridge National Laboratory, Oak Ridge, TN 37831, USA

**Keywords:** multilayer graphene, atmospheric CO_2_ mitigation, sustainability, multilayer graphene characterization

## Abstract

Graphene produced by different methods can present varying physicochemical properties and quality, resulting in a wide range of applications. The implementation of a novel method to synthesize graphene requires characterizations to determine the relevant physicochemical and functional properties for its tailored application. We present a novel method for multilayer graphene synthesis using atmospheric carbon dioxide with characterization. Synthesis begins with carbon dioxide sequestered from air by monoethanolamine dissolution and released into an enclosed vessel. Magnesium is ignited in the presence of the concentrated carbon dioxide, resulting in the formation of graphene flakes. These flakes are separated and enhanced by washing with hydrochloric acid and exfoliation by ammonium sulfate, which is then cycled through a tumble blender and filtrated. Raman spectroscopic characterization, FTIR spectroscopic characterization, XPS spectroscopic characterization, SEM imaging, and TEM imaging indicated that the graphene has fifteen layers with some remnant oxygen-possessing and nitrogen-possessing functional groups. The multilayer graphene flake possessed particle sizes ranging from 2 µm to 80 µm in diameter. BET analysis measured the surface area of the multilayer graphene particles as 330 m^2^/g, and the pore size distribution indicated about 51% of the pores as having diameters from 0.8 nm to 5 nm. This study demonstrates a novel and scalable method to synthesize multilayer graphene using CO_2_ from ambient air at 1 g/kWh electricity, potentially allowing for multilayer graphene production by the ton. The approach creates opportunities to synthesize multilayer graphene particles with defined properties through a careful control of the synthesis parameters for tailored applications.

## 1. Introduction

Graphene is a single-layer structure of polycyclic aromatic hydrocarbons [1]. The properties surrounding this material were speculated in the 1940s [1] and since its isolation in 2004 [2] have been investigated further [3]. Notably, reports have been made that the graphene structure has a high electron mobility (greater than 200,000 cm^2^V^−1^s^−1^) at electron densities of 2 × 10^−11^ cm^−2^, [1,2,4] displays the quantum Hall effect, [5,6,7,8,9] and possess a thermal conductivity range of 4.4 × 10^3^ to 5.78 × 10^3^ W/mK [9]. Furthermore, research reports have indicated that the graphene structure has a Young’s modulus of 1 TPa [1,2,10,11], an intrinsic strength of 130 Gpa [10,11], and can have a theoretical surface area of up to 2630 m^2^/g [12] and experimental surface area range of 200 to 700 m^2^/g [1,12,13].

The exact effect of these properties relates to the structural defects and functionalization of the graphene sheet [10,13], which are dependent on the method used to produce the graphene [3,10,13]. Different methods for graphene production include mechanical exfoliation, chemical vapor deposition, epitaxial growth, electrochemical exfoliation, pyrolysis, sonication, thermal exfoliation, liquid phase exfoliation, and chemical creation [3,10,13].

In this work, we describe a novel multilayer graphene synthesis process using CO_2_. This process differs from other CO_2_-based graphene synthesis methods in that the CO_2_ used in this process is directly sequestered from ambient air rather than originating from treated or purified CO_2_ stored in tanks. This allows for the design of continuous flow systems that enable the method to be scalable for commercial applications under optimum economy. Furthermore, the electrolytic recombination is done with graphene flakes in ethanol suspension compared to prior works, which exfoliate graphite rods using electrolysis. This method offers opportunities to recycle all of the generated chemical waste back into the process. This saves processing costs and allows for environmentally friendly method of production.

Variations in the production method can result in graphene predisposed to different structural defects and functionalization, which make it paramount to examine the properties of the multilayer graphene produced by this novel method.

The novel method for synthesizing multilayer graphene sheets from carbon dioxide involves the ignition of magnesium and carbon dioxide sequestered from air, followed by refinement in an aqueous suspension of exfoliated graphene [14]. The primary steps include (1) percolating a gaseous CO_2_ solution; (2) heating an aqueous monoethanolamine solution; and (3) collecting the released CO_2_ containing magnesium element for graphene production. The multilayer graphene product was characterized via Raman spectroscopy, scanning electron microscopy (SEM), Fourier transform infrared spectroscopy (FTIR), X-ray photoelectron spectroscopy (XPS), Brunauer–Emmett–Teller analysis (BET), and transmission electron microscopy (TEM). These analyses were used to study the number of layers present, the functionalization, and the surface area properties of the multilayer graphene product.

The novelty of this method is that it was designed as a scalable process to be readily applied to an industrial setting with no environmental waste rather than a laboratory setting with small-scale yields. The inventor of this method chose magnesium metal as opposed to other reducing metals such as sodium because the equipment available is more readily designed for higher combustion heats and is generally more stable than sodium. The inventor of this method also chose MEA to sequester carbon as opposed to other options—zeolites, metal–organic frameworks, mesoporous silicas, hydrotalcites, or activated carbons—because monoethanolamine (MEA) is easier to integrate into continuous flow systems. MEA also has the advantage of being continuously recycled, meaning that it is not wasted in the process. All other waste streams in the process are salts that can be dried or refined back to their original constituents and recycled into the proposed method using similar continuous flow systems. It should be noted that the goal of this research is to prove the efficacy of already patented technology, and not to dispute against other methods with hypothetical alternative chemicals.

## 2. Materials and Methods

**Figure 1:**
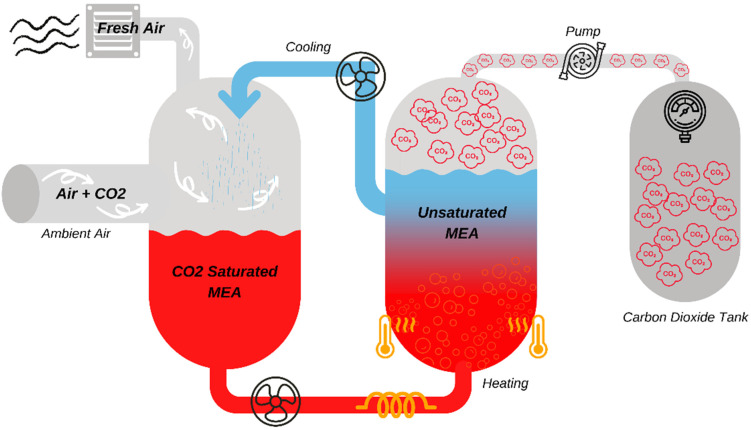
Process steps for isolation of CO_2_ in ambient air.

(1) Sequester of carbon dioxide gas from ambient air:

The sequester of carbon dioxide gas from ambient air comprises several steps, as shown in Figure 1. First the ambient air is percolated through an aqueous MEA solution at a low temperature (below 10 °C, below −50 °C, or below −80 °C). MEA is highly manageable when integrated into a supply line without chemical waste as a byproduct. Upon saturation of the MEA solution with carbon dioxide, the aqueous MEA-CO_2_ solution (30%) is heated to ~35 °C to release the adsorbed carbon dioxide, which is then stored in a collection tank.

**Figure 2:**
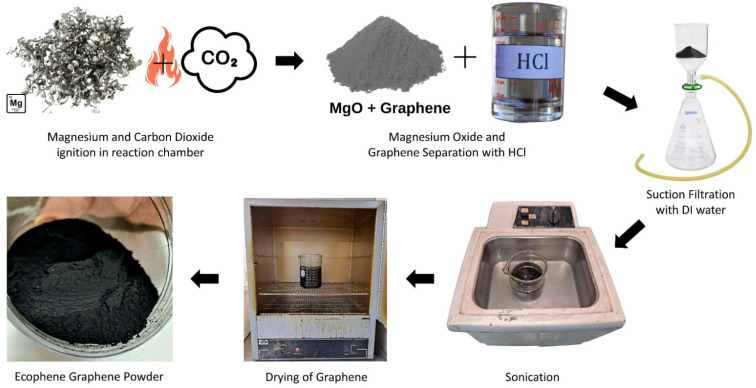
Process steps for sustainable synthesis of multilayer graphene from CO_2_ isolated from ambient air.

(2) Synthesis of multilayer graphene sheets from carbon dioxide gas:

The synthesis of multilayer graphene sheets from carbon dioxide gas comprises several steps, as shown in Figure 2. The isolated carbon dioxide is released in a reaction chamber containing magnesium, followed by ignition of the magnesium metal element in the presence of carbon dioxide to form magnesium oxide and graphene flakes according to the following reaction. Magnesium was chosen because the byproduct created is easier to recycle in a commercial setting, allowing for reuse into the production process.

(1) $${\mathrm{CO}}_{2}+2\mathrm{Mg}\to 2\mathrm{MgO}+C$$

Magnesium chloride, water, and graphene flakes are formed by washing the graphene flakes and magnesium oxide with aqueous hydrochloric acid solution, as shown in the following reaction.

(2) $$\mathrm{MgO}+C+2\mathrm{HCl}\;\to {\mathrm{MgCl}}_{2}+{\;H}_{2}O\;+C$$

The graphene flakes are then exfoliated in an ammonium sulfate solution (0.1 M with pH 6.5–7) and tumbled in a blender for several hours. Finally, the sheets are washed and dried.

(3) Analytical characterization of the multilayer graphene material:

Raman spectroscopy (Almad Raman TO-ARS-532, Thunder Optics—Montpellier, France) was performed with a 532 nm laser with a resolution of 8 cm^−1^ and a 20 × 40 N microscopic objective. The laser power had a variable range of 200 mW and readings, which were taken at approximately 175 mW. The wave numbers measured were 300 cm^−1^ to 4000 cm^−1^. Only the range relative to characterizing graphene is presented. The ATR-IR spectra of the multilayer graphene were measured with a FTIR spectrometer (Spectrum 400 FT-IR/NIR spectrometer, PerkinElmer—Waltham, MA, USA) with a diamond ATR attachment allowing a spectral resolution of 2 cm^−1^ in the range of 4000–600 cm^−1^. X-ray photoelectron spectroscopy (XPS, Model K-Alpha, ThermoFisher Scientific—Waltham, MA, USA) was performed. Wide-energy range survey spectra (0–1350 eV) were acquired for qualitative and quantitative analysis (pass energy = 200 eV; step size = 1.0 eV). An assessment of the chemical bonding of the identified elements was accomplished by collecting core-level spectra over a narrow energy range (pass energy = 50 eV; step size = 0.1 eV). SEM (S-3400 N, Hitachi—Chiyoda City, Tokyo, Japan) was performed. BET (NOVA Touch LX2, Quantachrome— Boynton Beach, FL, USA) analysis was performed. For the initial gas desorption, the samples were heated to 200 °C at a heating rate of 5 °C/min over a period of 360 min. The absorbing gas was nitrogen (cross-sectional area of 16.2 Å2/molecule). The absorption and desorption curves were obtained from the unit and the samples were cooled with liquid nitrogen. The porosity of the sample was calculated using non-local density functional theory with slit pores for nitrogen gas on carbon. Scanning transmission electron microscope analysis (UltraSTEM 200, Nion—Kirkland, WA, USA) was performed with a field emission gun (FEG) operating at 100 kV to demonstrate the number of layers of graphene.

## 3. Results and Discussion

**Figure 3:**
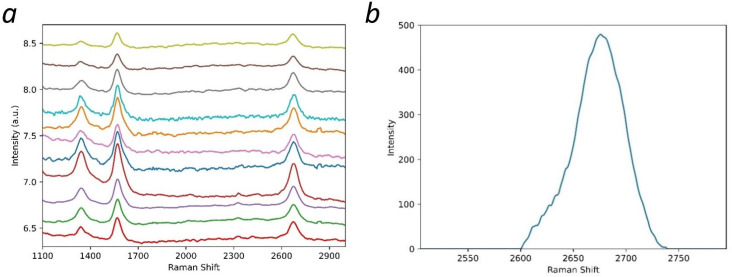
(**a**) Raman spectra for eleven random samples of the multilayer graphene powder and (**b**) 2D peak after baseline removal.

Figure 3a shows the Raman spectra obtained for the product. Eleven Raman spectra were obtained from random samples of the multilayer graphene powder; the spectra show no deviation from one another as is expected in a uniform product. Three distinct peaks are seen for each spectrum. The peak at ~1340 cm^−1^ denotes the D peak; the peak at ~1571 ± 2 cm^−1^ denotes the G peak; and the peak at ~2674 ± 3 cm^−1^ denotes the 2D peak. The D peak was not analyzed beyond recognizing that its presence indicated that structural disorders were present in the multilayer graphene [15,16,17]. Both the locations and intensity ratio of the G peak and 2D peak were determined. The locations and intensity ratio of these two peaks have been used in other reports to determine the number of layers of graphene [16,17,18,19,20,21]. Furthermore, the shape of the 2D peak is indicative of the number of layers of graphene [16,17,18,19,20,21]. In Figure 3b, one of the 2D peaks from the spectra is shown after baseline removal. This peak can be seen to be a composite of multiple Lorentzian peaks that form into two distinguishable peaks (at ~2674 cm^−1^ and at ~2635 cm^−1^). The presence of this sub-peak indicates that the multilayer graphene has more than ten layers [16,17], but the exact number of layers cannot be determined from the shape of the 2D peak after a certain threshold [16,17].

From Figure 3b, the average intensity ratio (I_G_/I_2D_) was found to be 1.28. Previous work has modeled intensity ratio as a function of the number of graphene layers using an exponential equation; as the number of layers approaches infinity, the limit of this equation is 1.33 [18]. Due to experimental variance, this makes any measured intensity ratio at or around this value only viable for indicating that the sample has greater than five layers. Thus, the Raman spectra indicate that structural disorders and possible functionalization of the multilayer graphene were present, with the shape of the 2D peak, indicating that the sample was composed of more than ten layers.

**Figure 4:**
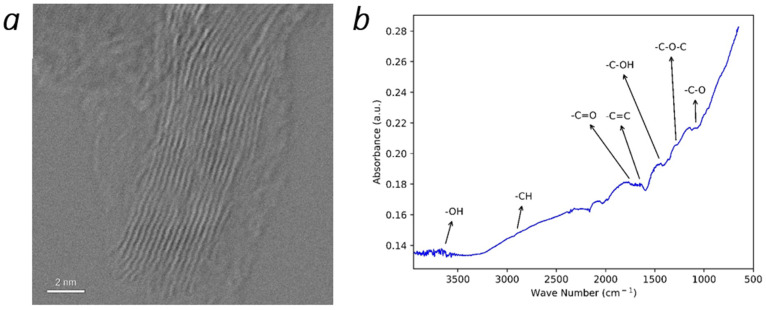
(**a**) TEM image and (**b**) FTIR spectra of the synthesized multilayer graphene.

In Figure 4a, a TEM image of the multilayer graphene sample is presented. The results of this test indicate that the multilayer graphene is composed of fifteen to sixteen layers. These results agree with the analysis from Raman spectra. FTIR spectrum of the graphene sample is shown in Figure 4b, and the functional groups are labeled. The peak at ~2300 cm^−1^ is assigned to CO_2_. The broad peak around 3630 cm^−1^ is attributed to -OH stretching [22]. The -C-H, -C=O, and C=C stretching were observed at 2900, 1750, and 1650 cm^−1^, respectively [23]. Bands at 1450, 1285, and 1085 cm^−1^ were associated with C–O in carboxyl or hydroxyl groups, -C-O-C, and C-O groups [24]. These results suggested that the oxygen molecules present in the graphene, and functional groups were introduced to graphene during the synthesis. The presence of the functional groups, such as -OH and -COOH, could help its dispersion in solvents and matrixes, and thus make it a potential material for different applications.

**Figure 5:**
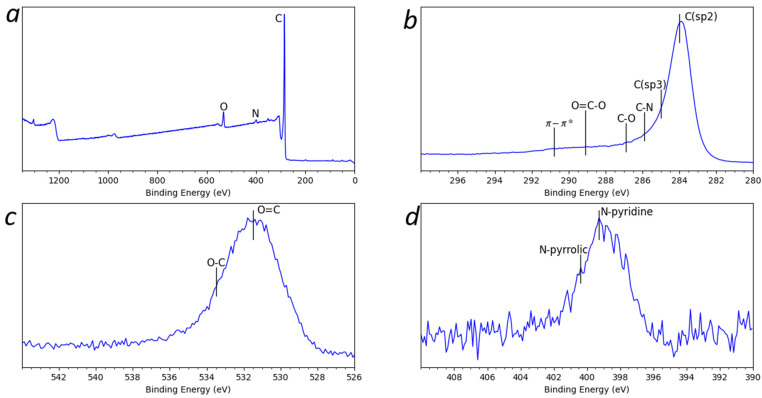
(**a**) XPS spectra analysis of (**b**) C1s, (**c**) O1s and (**d**) N1s for the synthesized multilayer graphene.

XPS was conducted to further confirm the chemical composition of the graphene. As shown in Figure 5, C, O, and N were observed in the graphene [25,26]. High resolution scan of C1s (Figure 5b) revealed that C (sp2, 284.6 eV)), C (sp3 (285.1 eV), C-O (287.1 eV), O=C-O (288.6 eV), C-N (286.8 eV), and p-p* (291 eV) existed in the graphene. Similarly, C=O (531.6 eV) and C-O (532.9 eV) were observed in O1s (Figure 5c). N exists in the form of Pyridine- (398.6 eV) and pyrrolic-N (400.5 eV), as shown in Figure 5d. These results confirmed the introduction of the functional groups during the synthesis.

**Figure 6:**
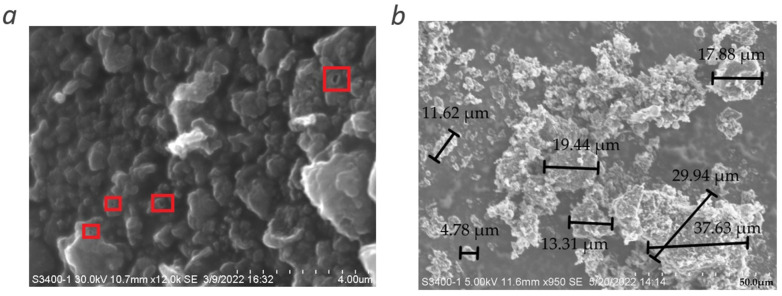
SEM images of (**a**) the surface and (**b**) the flake of the synthesized multilayer graphene.

In Figure 6a, an SEM image of the surface of the multilayer graphene sample flakes is shown. This surface is not smooth, which is expected as the large flakes are composed of smaller flakes of graphene with fifteen layers in different orientations. Some aberrations correlate with the functionalization observed from the FTIR spectra. The locations in Figure 6a, indicated by red boxes, show holes on the surface. These are likely due to oxygen or nitrogen bonds in the aromatic structure, surface bonded oxygen, or carbon bonded as alkanes. In Figure 6b, several of the multilayer graphene flakes from the sample are shown. The diameter of some of the flakes is marked in the image. The flakes were measured in sizes ranging from approximately 2 µm to 80 µm.

**Figure 7:**
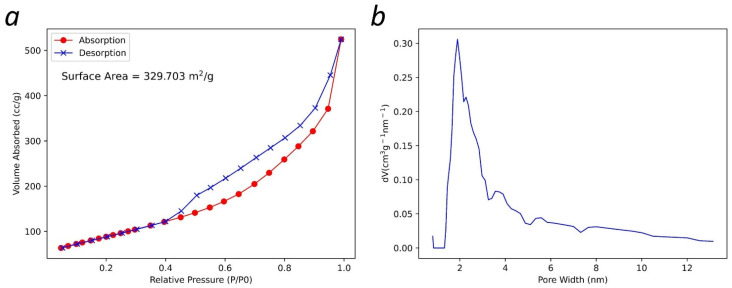
(**a**) BET isotherms and surface area characterization; and (**b**) BET pore size distribution of the multilayer graphene.

In Figure 7a, the absorption and desorption isotherms for the multilayer graphene sample are shown. BET analysis measured a surface area of ~330 m^2^/g. Since the presence of the oxygen functional groups were shown in the FTIR spectrum, a comparison to the measured surface areas of reduced multilayer graphene oxides in other studies [13,27,28,29,30] indicates that the synthesized multilayer graphene is comparable in nature. In Figure 7b, the pore distribution for the multilayer graphene (assuming a slit pore) is shown. Approximately 51% of the pores are distributed between 0.8 nm to 5 nm.

**Commercial viability analysis of the synthesis process**: The greatest limiting factor in the process is the electrical requirement per material input. At 400 ppm of carbon dioxide in ambient air, it is estimated that approximately 64 cubic meters of air would need to be processed per gram of carbon. At an Ecophene facility, with a production capability of 4.5 kg per month, the current electrical requirements from sequestered carbon dioxide in ambient air to multilayer graphene powder require about 1 kwh per gram of multilayer graphene. This data is for equipment having a maximum output of up to 10 kg per month; thus, with larger equipment and more sophisticated autonomous throughput systems, the electrical cost per gram is expected to decrease significantly. The method used reduced labor costs since a less-skilled workforce is required for operation and the potential for automation labor costs can almost be eliminated. Finally, the current production system has a relatively high material loss of ~3%. With more sophisticated systems, this material loss can be significantly reduced. The combination of these factors means that multilayer graphene can be reliably produced at about 20–25% cheaper than the market value of $70/g.

**Environmental Implications:** The novelty of the method used demonstrates a practical method of converting atmospheric carbon dioxide, the primary greenhouse gas responsible for anthropogenic climate change, into useful raw material with various benefits. Although it may not be enough to remove all of the excess carbon dioxide responsible for anthropogenic climate change, it demonstrates that atmospheric carbon dioxide mitigation can be achieved in a cost-effective and environmentally beneficial way. With further investigation, the production cost could be further reduced with quality improvement of the material.

## 4. Conclusions

Eleven Raman spectra of the multilayer graphene sample obtained from different points were presented, and a view of the 2D peak from one of the spectra after baseline removal was shown. Analysis of the data indicated that the multilayer graphene sample composed of more than ten layers, structurally disordered and functionalized. The TEM image of the multilayer graphene indicated a thickness of 15–16 layers. The FTIR spectra showed the presence of C-O-C, -C=O, C-O, and OH functional groups in the sample; the XPS spectra confirmed the presence of these functional groups as well as nitrogen-containing groups (pyridine and pyrrolic-N). These were introduced during their synthesis. The absorption-desorption isotherm of the multilayer graphene indicated a measured surface area of ~330 m^2^/g, and the pore distribution indicated ~51% of the pores between 0.8 nm and 5 nm in diameter. This work demonstrates a novel and scalable synthesis method to produce multilayer graphene from CO_2_ at 20–25% lower cost/gram with various physiochemical features that can be potentially tailored for a wide range of applications. Additionally, this synthesis method was developed to have a net-positive impact on the environment. The recovery of CO_2_ from ambient air reduces greenhouse gases in the atmosphere, and this reduction contributes to curtailing climate change. Furthermore, the chemical waste generated by the process can be recovered and recycled; this lends itself to the sustainability of the method.

## 5. Patents

Multilayer graphene used in this manuscript is based on the patented technology. Alvarez, L. “Apparatus, System and Method for Conversion of Atmospheric Carbon Dioxide to Graphene”. United States Patent Number: US 11,192,790 B2, 7 December 2021.

## Data Availability

Not applicable.
